# Ju-LiteMobileAtt: A lightweight attention network for efficient jujube defect classification

**DOI:** 10.1371/journal.pone.0337898

**Published:** 2025-12-02

**Authors:** Xiyuan Zhu, Hongtao Dang, Xiaoyuan Jin, Xun Li

**Affiliations:** Xijing University, Xi’an, Shaanxi, China; Hohai University, CHINA

## Abstract

Surface defect detection of organic jujubes is critical for quality assessment. However, conventional machine vision lacks adaptability to polymorphic defects, while deep learning methods face a trade-off—deep architectures are computationally intensive and unsuitable for edge deployment, whereas lightweight models struggle to represent subtle defects. To address this, we propose Ju-LiteMobileAtt, a high-precision lightweight network based on MobileNetV2, featuring two key innovations: First, the Efficient Residual Coordinate Attention Module (EfficientRCAM) integrates spatial encoding and channel interaction for multi-scale feature capture; Second, the Cascaded Residual Coordinate Attention Module (CascadedRCAM) refines features while preserving efficiency. Experiments on the Jujube12000 dataset show Ju-LiteMobileAtt improves accuracy by 1.72% over baseline while significantly reducing parameters, enabling effective real-time edge-based jujube defect detection.

## 1. Introduction

As an ecologically adaptable and economically valuable crop, the jujube tree is widely cultivated in arid and semi-arid regions globally. Its primary production regions span India, the Middle East, China, Southern Europe, and North Africa. In India’s arid zones (e.g., Rajasthan and Maharashtra), the dominant “Ber” variety is primarily consumed fresh and processed locally [1]. In the Middle East (e.g., Iran and Saudi Arabia), the jujube tree is termed “desert gold” due to its centuries-old cultivation tradition. The drought-resistant “Medjool” variety dominates, with fruits primarily dried or used in traditional medicine. This region accounts for over 28% of global dried jujube production annually. FAO data indicates Palestine as a major Middle Eastern jujube producer, contributing significantly to regional output. Large-scale cultivation in China’s Xinjiang and Shandong provinces, alongside Spain and Italy in Southern Europe, further underscores its economic importance in global specialty agriculture. The fruits’ nutritional value and versatile applications drive ongoing growth in the global jujube industry.

As an important characteristic agricultural product in China, jujube requires accurate surface defect detection, which directly impacts market acceptance of end products and is closely tied to processing efficiency and farmers’ economic benefits. Developing efficient and reliable defect detection technology is critical to advancing the jujube industry’s transition toward automated sorting and standardized grading. However, current detection methods still struggle with complex industrial interferences such as light fluctuations, mixed varieties, and tiny defect recognition, failing to meet the high-throughput and high-precision demands of large-scale production.

Traditional machine learning methods were the dominant approach for jujube surface defect detection in early research. These methods relied on manually engineered features (e.g., color histograms [2], LBP texture, edge gradients) and classifiers such as SVM [3] and random forest. While effective for basic classification in controlled environments, they exhibited critical limitations in handling the complexity of jujube defects: heavy reliance on handcrafted features prevented adaptability to polymorphic defect variations, feature overlaps caused poor cross-scenario generalization capabilities, classifiers exhibited sensitivity to weak signal detection [4] and data imbalance, leading to high false negatives, low accuracy, and inadequate handling of complex defects and environmental noise, and the models lacked scalability, were prone to overfitting in small datasets, and unable to meet the dynamic, large-scale detection demands of the jujube industry.

In recent years, advancements in machine vision technology have revolutionized agricultural product quality evaluation. The rapid development of deep learning has further opened new possibilities for jujube surface defect detection. Convolutional Neural Networks (CNNs) have exhibited exceptional performance in image-based defect detection and classification tasks [5], leveraging their robust feature extraction and non-linear representation capabilities to enable widespread adoption across diverse image recognition applications. Additionally, deep learning-based detection technologies have gained industry recognition and are increasingly deployed in industrial settings. Integrating these algorithms into automated jujube defect classification processes enhances product standardization, reduces defect rates, and achieves near-optimal detection performance even in resource-limited devices and environments [6].

To address these challenges, this paper proposes an optimized lightweight jujube defect classification network, Ju-LiteMobileAtt. The network incorporates a coordinate attention mechanism initialized with zero to prioritize weak defect signal learning, enhancing focus on subtle defects and reducing missed detections while improving accuracy. Additionally, the EfficientRCAM module employs depth-wise convolutions and residual connections to strengthen defect feature propagation, reduce parameters, prevent subtle information loss, and stabilize performance. A CascadedRCAM module is further designed to simplify the network’s tail structure by compressing channels, eliminating redundant features, and emphasizing key defect characteristics, thereby balancing accuracy and efficiency. Finally, attention modules are embedded at the end of each stage to selectively focus on defects across different scales, enhancing multi-scale feature extraction and mitigating classification bias.

The main contributions of this paper are as follows:

(1) To address the problem that low-contrast defects on jujube surfaces are easily ignored, a “channel-spatial” two-stage collaborative attention mechanism is proposed. It first strengthens defect-related feature channels and then focuses on defect locations to improve the recognition ability for such defects.
(2) The Efficient Residual Coordinate Attention Module (EfficientRCAM) is designed. Targeting the fine-grained characteristics of jujube defect features, it uses depth-wise convolutions to accurately capture defect details and avoids loss of subtle defect features during propagation through residual connections. While strengthening the transmission of jujube defect features, it reduces redundant parameters and improves the model’s ability to distinguish complex defects.
(3) The CascadedRCAM module is designed, which builds a cascaded structure based on EfficientRCAM. It integrates multi-scale defect information of jujubes through “residual fusion of enhanced features and original features”, achieving cross-scale feature complementarity. This design simplifies computation while retaining key features of jujube defects, balancing detection accuracy and real-time performance, and adapting to the efficiency requirements of jujube sorting scenarios.
(4) A selective attention embedding strategy is adopted, where attention modules are only added at the last layer of each stage. By targeting the multi-scale characteristics of jujube defects, these modules can adaptively enhance the weights of defect features extracted at each stage, improve the model’s targeted extraction ability for jujube defects at different scales, and reduce classification bias caused by scale differences.

The remaining parts of this paper are organized as follows: Section 2 summarizes related work on jujube surface defect classification. Section 3 introduces Ju-LiteMobileAtt and its related improvement strategies. Section 4 describes experimental details. Section 5 analyzes experimental results. Section 6 summarizes the main achievements of this paper.

## 2. Related work

### 2.1. Feature enhancement methods for low-contrast defects

To solve the problem of feature enhancement for low-contrast defects on jujube surfaces, researchers have proposed various methods with respective limitations:

Jiang et al. [7] proposed JujubeNet, which improves classification efficiency and lightweight performance through multi-branch convolutions and CBAM attention mechanisms but lacks precision in fusing features for extremely subtle defects. Wen et al. [8] introduced an improved residual network that optimizes gradient propagation and detection efficiency but exhibits poor adaptability to low-contrast defects caused by severe illumination variations. Ju et al. [9] developed a transfer learning framework that enhances model stability on small datasets, yet suffers from limited cross-domain generalization capability due to domain discrepancies between generic and defect-specific features. Zakeri et al. [10] combined computer vision techniques with classifiers to achieve high accuracy, though manually engineered features demonstrate weak robustness against low-contrast defects in complex backgrounds. Zeng et al. [11] proposed a hybrid CNN-Vision Transformer model maintaining 97% accuracy under 30% brightness increases and 93% accuracy under 30% brightness decreases for plant disease detection. However, this model’s increased parameter count imposes higher hardware requirements.

To address these issues, this paper proposes the following improvements: introducing the EfficientRCAM module to enhance the capture of subtle defect features on jujube surfaces.

### 2.2. Classification models for multi-class defects

To address the problem of class confusion same defect under different varieties and maturity levels, related studies have improved model generalization ability through various methods, each with advantages and disadvantages:

Nirere et al. [12] integrated hyperspectral imaging with SVM and LS-SVM classifiers, optimizing features through preprocessing to offer foundational insights for agricultural defect classification. However, hyperspectral equipment’s high cost and complexity limit its practical adoption. Rahimi et al. [13] leveraged machine vision to extract multidimensional features, combining them with data mining techniques after screening to validate feature selection and mining efficacy. Despite this, their manual feature design approach limits adaptability to complex defect patterns. Luo et al. [14] employed wavelet transform for preprocessing and fused multi-source features to enhance SVM model performance, demonstrating fusion’s utility in distinguishing complex defects. However, the method remains sensitive to low-contrast defects and incurs high computational demands. Li et al. [15] proposed a Transformer-based multi-modal fusion framework to improve grape disease detection accuracy but struggles to distinguish early-stage symptoms of downy mildew and spot blight.

To address these issues, this paper proposes the following improvement plan: the proposed CascadedRCAM enhances hierarchical aggregation of features for multi-scale defects through multi-stage attention progressive strengthening.

### 2.3. Lightweight models

In research on adapting lightweight models for jujube detection to industrial deployment, related methods have respective advantages and disadvantages:

Liu et al. [16] proposed an improved CA-MobileNetV3, replacing the SE module with coordinate attention and leveraging transfer learning to enhance positional and feature correlation capture, thereby improving generalization. However, its medical scenario design requires validation for jujube defect specificity. Mugada and Lakshmi [17] developed DMEGrade-Net based on MobileNet, employing dilated convolutions to strengthen high- and low-level feature extraction and adaptive transfer learning for grading optimization. Yet, its multi-stage architecture may increase computational complexity, compromising real-time performance. Yuan et al. [18] introduced MobileNetV2-SE, combining pseudo-labels to achieve lightweight accuracy maintenance. However, the SE module demonstrated limited focusing capability on low-contrast jujube defects. Huang et al. [19] designed a Transformer network integrating spectral-spatial self-attention with lightweight components like depthwise separable convolutions, enabling global spectral-spatial feature extraction for hyperspectral image classification enhancement.

To address the lightweight requirements of jujube surface defect detection, this study proposes the Ju-LiteMobileAtt model. This architecture inherits the lightweight framework of MobileNetV2 and incorporates EfficientRCAM, CascadedRCAM, and cascaded residual coordinate attention mechanisms to reduce computational costs while preserving critical defect features.

## 3. Methods

### 3.1. Selection of framework network

To select a benchmark network suitable for jujube defect detection, this study conducted comparative experiments on various mainstream models using the self-constructed Jujube12000 dataset [20]. Evaluated architectures included traditional convolutional neural networks (e.g., MobileNetV2, MobileNetV3, ResNet, ShuffleNetV2, EfficientNet, RepViT) and the Transformer-based MobileViT. Experimental results demonstrated that MobileNetV2 achieved the highest classification accuracy of 97.1%. Further analysis indicated that while the Transformer architecture excels in modeling long-range feature correlations, it lacks capability in capturing subtle image details. In jujube defect detection, local features—such as pericarp texture variations and minute color differences—are critical for classification outcomes, rendering MobileViT less suitable for this task.

To evaluate model performance, experiments were conducted on the Jujube12000 dataset [20]. ResNet achieved 96.43% accuracy with 11.18M parameters, while EfficientNet and MobileNetV3 demonstrated higher accuracy (97.93% and 97.56%, respectively) but required 4.02M and 4.21M parameters. Though these models exhibited strong classification performance, their large parameter counts hindered device deployment. Lightweight alternatives such as ShuffleNetV2 (95.96% accuracy, 3.5M parameters), MobileViT (96.93%, 0.95M parameters), and RepViT (96.23%, 2.17M parameters) showed reduced parameter requirements but compromised accuracy. Notably, RepViT and similar models failed to meet precision standards for jujube defect classification due to their trade-off between parameter efficiency and classification performance.

In summary, MobileNetV2 demonstrated outstanding comprehensive performance with a classification accuracy of 97.1% and only 2.23M parameters, achieving a balanced trade-off between detection accuracy and model efficiency. Based on these results, we selected MobileNetV2 as the benchmark framework and proposed the Ju-LiteMobileAtt model, which combines lightweight architecture with high precision. This enhanced model significantly improved jujube defect classification accuracy while reducing parameter counts through structural optimization, accelerating inference speed, and enabling efficient deployment in industrial-scale production scenarios.

### 3.2. MobileNetV2

**Fig 1:**
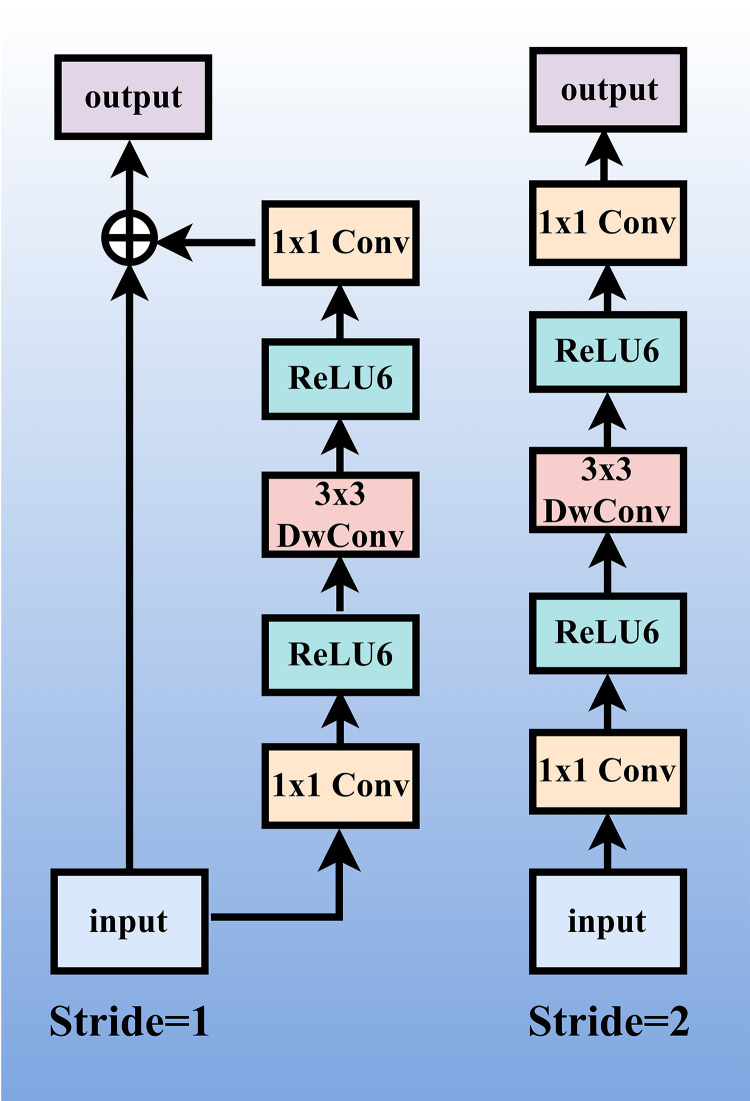
The detailed structure of MobileNetV2.

MobileNetV2, proposed by Sandler et al. in 2018, is a lightweight deep neural network designed for resource-constrained environments such as mobile terminals and embedded devices. Its core objective is to achieve a performance-efficiency trade-off through structural reorganization and convolution optimization. The architecture employs depth-wise separable convolutions, which decompose traditional convolutions into depth-wise and point-wise operations, reducing total parameters by nearly 90% and computational complexity by approximately 30% while maintaining classification accuracy comparable to conventional models on large-scale datasets like ImageNet. Additionally, MobileNetV2 introduces inverted residual blocks with linear bottlenecks to mitigate information loss in low-dimensional spaces and enhance feature transmission efficiency. The architecture further supports flexible model scaling via width and resolution multipliers, enabling adaptation to diverse device computational capabilities. These features make it particularly suitable for real-time inference on mobile platforms, offering a practical and scalable solution for efficient visual processing under resource constraints. Fig 1 illustrates the detailed architecture of MobileNetV2.

### 3.3. CoordAtt attention mechanism module

The CoordAtt attention mechanism proposed in this paper integrates the cascaded structure of the channel attention module SE [21] and directional attention module ECA [22], specifically tailored for jujube defect classification. It enables precise detection of defects such as cracks, mildew, and bird pecks through a two-stage feature filtering process. Structurally, CoordAtt first performs defect-background separation in the channel dimension using the SE module, directly addressing the core challenge of complex texture interference in jujube defect classification. The SE module focuses on channel response differences between normal jujube surface textures and defect features. By amplifying high-response channels in mildewed regions and suppressing redundant channels from normal pericarp textures, the mechanism reduces misclassification caused by variety-specific textures during mixed inspections, thereby providing cleaner channel features for subsequent classification stages.

Based on the SE module, the CoordAtt cascades an ECA module to achieve spatial defect localization, primarily addressing the classification of minor defects. The ECA module employs grouped convolutions to enhance perception of direction-sensitive features, precisely capturing spatial patterns such as linear crack distributions along the jujube’s longitudinal axis and localized bird-peck aggregations.

The progressive channel screening and direction enhancement mechanism of CoordAtt aligns well with the multi-dimensional characteristics of jujube defects. The SE module addresses channel-level redundancy, while the ECA module resolves spatial ambiguity. Their combined effect enables the model to simultaneously focus on defect-related channels during classification and achieve precise spatial localization. This mechanism is particularly effective for scenarios involving jujubes with complex surface textures and diverse defect morphologies. Additionally, CoordAtt preserves original feature integrity through residual enhancement, and its computational process is defined as:


$$F_{coordatt}=[\sigma (AvgPool(GroupConv(s⨀F;K,g),dim=\text{1}))⨀(s⨀F)]+F$$ (1)

**Fig 2:**
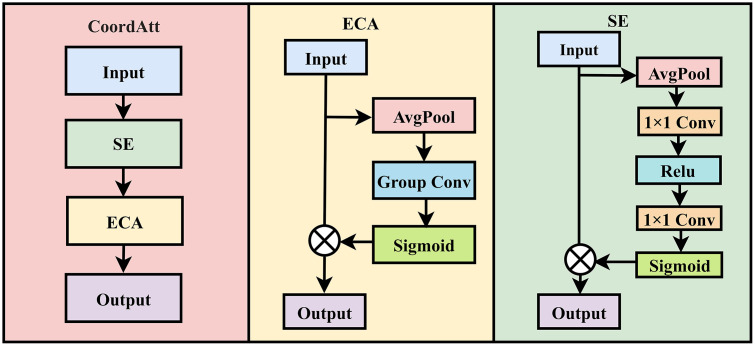
The Overview of the proposed CoordAtt module.

Where $s=\sigma (FC(ReLU(FC(AvgPoold(F);W_{\text{1}}));W_{\text{2}}))$. Fig 2 illustrates the SE module, ECA module, and CoordAtt module respectively.

### 3.4. EfficientRCAM module

The EfficientRCAM module is specifically designed for detecting surface defects on jujube fruits. By deeply integrating a lightweight architecture with an enhanced coordinate attention mechanism, it significantly improves the capture of tiny, low-contrast defects while controlling computational costs. The module adopts a depth-wise separable convolution architecture through the ConvBNHSwish module, utilizing 3 × 3 depth-wise convolutions to retain linear features of cracks, block-like features of mold, and edge details of multi-scale defects, thereby adapting to the complex surface textures and diverse defect morphologies of jujube fruits. To enhance defect area localization precision, it incorporates a CoordAtt sub-module. Key improvements include lightweight channel compression pathways and attention synergy, enabling clearer distinction between the gray-level differences of initial mold on jujube surfaces and normal pericarp textures.

In addition, the optimization of the module in terms of training stability and nonlinear expression is significant: The convolution layer for generating attention weights in CoordAtt adopts zero initialization, making the module approximately an identity mapping in the early training stage, effectively alleviating the gradient dispersion problem of deep networks; while the full embedding of the H-Swish [23] activation function enhances the nonlinear expression ability:


$$H-Swish(x)=x\cdot \frac{RELU\text{6}(x+\text{3})}{\text{6}}$$ (2)

Where Relu6=min(max(0,x),6).

**Fig 3:**
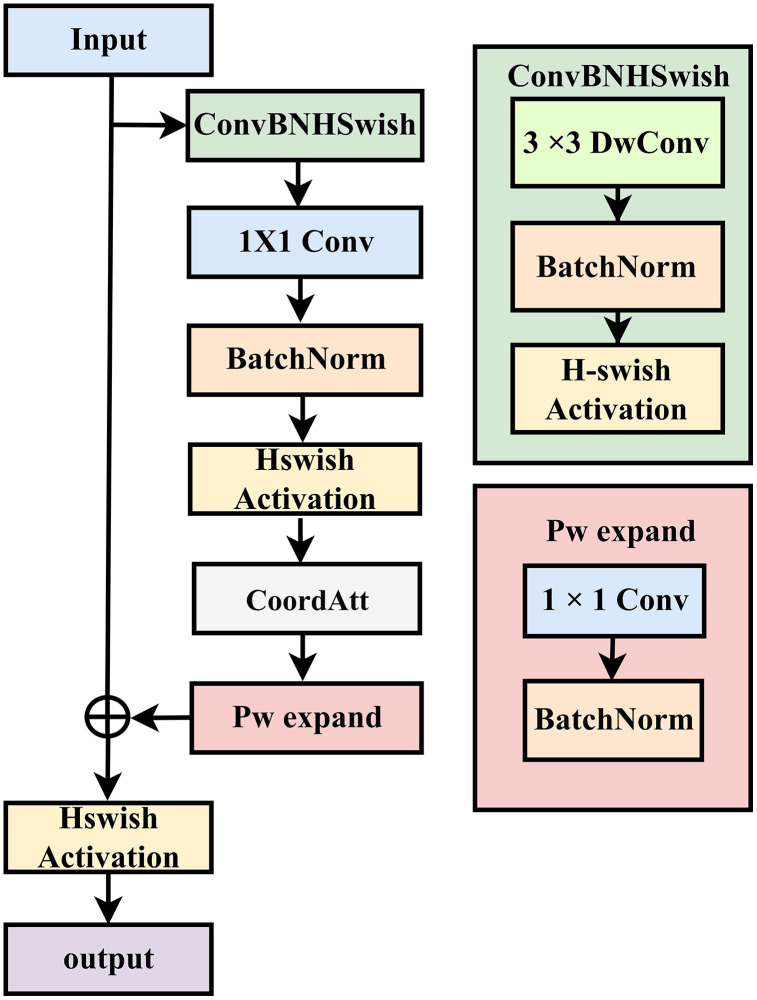
The structure of the EfficientRCAM module.

Compared with general lightweight modules, the targeted optimizations of EfficientRCAM are reflected in three key aspects: depth-wise convolutions’ multi-scale receptive fields accommodate the size variations of jujube defects; CoordAtt’s dual attention mechanisms simultaneously suppress complex background interference and enhance defect features; and the lightweight channel compression strategy balances feature representation with computational efficiency. Fig 3 illustrates the structure of the EfficientRCAM module.

### 3.5. CascadedRCAM module

The CascadedRCAM module employs a three-layer architecture comprising single-module enhancement, residual fusion, and channel adaptation to address the practical requirements of jujube surface defect detection. It directly adopts EfficientRCAM units as core enhancement components, replacing the conventional multi-module cascading approach [24]. Compared with traditional cascading schemes, it reduces the number of parameters and mitigates redundant feature accumulation, ensuring efficient feature extraction while retaining the capability to detect multi-scale defects.

To address the large-scale variation challenge in jujube defect detection, the module establishes residual connections between enhanced and original features. Original features preserve low-level details, while EfficientRCAM-enhanced features emphasize high-level semantics. Their fusion achieves cross-scale information complementarity, ensuring balanced detection of defects across varying sizes. In channel processing, the module employs 2x channel compression via the ConvBNHSwish block, retaining more intermediate features than conventional 4x compression. This strategy is critical for detecting low-contrast defects in jujube images, as it preserves weak texture details and establishes a coherent enhancement pathway. Non-linear operations within the H-Swish activation function and EfficientRCAM module further amplify perceptual capabilities in scenarios where grayscale differences between defects and normal pericarp are minimal.

Its processing flow can be expressed as:


$$F\_out=ConvBNHSwitc\text{h}(F_{in}+F_{rcam},K_{adapt},C/\text{2})$$ (3)

$$\begin{array}{c} {\text{F}}_{rcam}=[\sigma \left(AvgPool\left(GroupConv(\text{s}⊙DepthConv({\text{F}}_{in};{\text{K}}_{depth});{\text{K}}_{g},g),dim=\text{1}\right)\right)⊙\\ \;\;\;\;(\text{s}⊙DepthConv({\text{F}}_{in};{\text{K}}_{depth}))]+DepthConv({\text{F}}_{in};{\text{K}}_{depth}) \end{array}$$ (4)

Where $s=\sigma \left(FC\left(ReLU\left(FC(AvgPool(Dept\text{h}Conv(F_{in};K_{dept\text{h}}));W_{\text{1}})\right);W_{\text{2}}\right)\right)$.

### 3.6. Ju-LiteMobileAtt

Based on the MobileNetV2 architecture, this paper constructs a lightweight classification model with high accuracy to meet the practical demands of jujube surface defect detection. This is achieved through the integration of the CoordAtt composite attention mechanism (composed of cascaded SE and ECA modules) and the innovative design of EfficientRCAM and CascadedRCAM modules. The model retains MobileNetV2’s lightweight advantages via its inverted residual structure and depth-wise separable convolutions, while enhancing performance through a three-tiered feature optimization mechanism.

Specifically, the CoordAtt module first screens defect-related key channel features via SE channel attention and subsequently enhances direction-sensitive spatial features. The EfficientRCAM module extracts fine-grained textures through depth-wise convolutions, amplifies feature signals of tiny defects using attention enhancement and channel expansion, and incorporates residual connections to minimize information loss during feature propagation. The CascadedRCAM module strengthens global contours of large-area defects while preserving weak features (e.g., subtle grayscale variations in early-stage mold) through cross-scale residual fusion of enhanced and original features, thereby improving the model’s recognition stability in complex scenarios.

**Fig 4:**
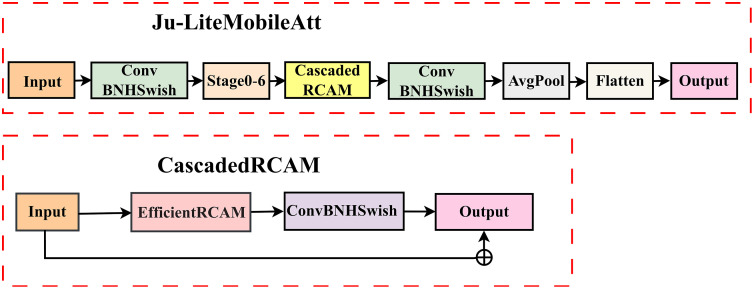
The overall network structure and core module design.

The model employs a selective embedding strategy, deploying CoordAtt modules exclusively at the final layer of each stage. This approach mitigates parameter redundancy from full network embedding while selectively enhancing the discriminative capacity of stage outputs. The design maintains a parameter count of 2.16 million while achieving 98.82% defect classification accuracy, striking an optimal balance between lightweight architecture and detection precision. Fig 4 depicts the network architecture and core modules, while Table 1 details inverted residual configurations across seven stages.

**Table 1:** Shows detailed information of inverted residuals in seven stages.

Stage	Block Count	Input Channels	Output Channels	Expand Ratio	Stride (First/Subsequent Blocks)	CoordAtt Applied At
**Stage0**	1	32	16	1	1/-	None
**Stage1**	2	16	24	6	2/1	2nd block
**Stage2**	3	24	32	6	2/1	3rd block
**Stage3**	4	32	64	6	2/1/1/1	4th block
**Stage4**	3	64	96	6	1/1/1	3rd block
**Stage5**	3	96	160	6	2/1/1	3rd block
**Stage6**	1	160	320	6	1/-	1st block (Yes)

## 4. Research methods

### 4.1. Dataset description

**Fig 5:**
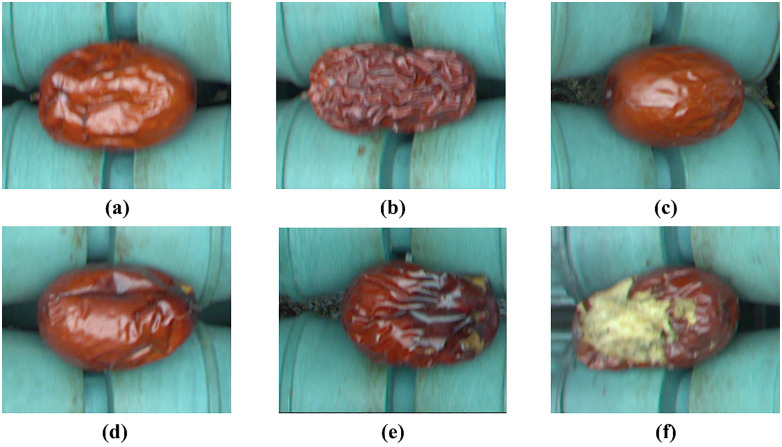
Six types of samples: (a) Deformed, (b) Wrinkled, (c) Normal, (d) Cracked, (e) Mold, (f) Bird-Pecked.

The dataset used in this paper comes from the “Jujube12000” dataset collected and created by Jiang et al. It contains 10,000 defect images across 5 surface defects (2,000 images for each defect) and 2000 images of normal jujubes. The Jujube12000 dataset includes six categories: wrinkles, deformation, mold, cracks, bird pecks, and normal jujubes. Fig 5 shows some sample images.

### 4.2. Data augmentation

During model training, images are typically processed in batches, which requires consistent sizes across each batch—making image size standardization essential before training. Despite potential object deformation, adjusting all images to the same size balances training speed and accuracy for large datasets. Given that multiple studies confirm 224 × 224 pixels optimizes the trade-off between model accuracy and computational efficiency, this study standardized jujube images to 224 × 224 pixels for training. Additionally, data augmentation was applied to improve sample quality and model generalization, with strategies detailed below.

Random cropping: This method expands data by randomly cropping local regions from original images (scaled to 70%−100% of the original size, aspect ratio 0.8–1.25) and standardizing them to 224 × 224 pixels. Bicubic interpolation ensures scaled image clarity, encouraging the model to focus on diverse local details to enhance adaptability to local changes, stabilize the model, and reduce overfitting.

Flipping: Random horizontal and vertical flipping (50% probability each) increases sample diversity without altering core image content. This exposes the model to images in different directions during training, building adaptability to direction changes and improving robustness.

Comprehensive augmentation: Combined transformations simulate real-scenario image variations to further boost generalization: 1) Rotation and shearing: Random rotation within ±15° and shearing within 10° to mimic morphology changes from deviated shooting angles, aiding adaptation to target features from different perspectives; 2) Random occlusio: Random rectangular regions (2%−20% of the image) with pixels replaced by preset values, enhancing recognition of partially occluded targets; 3) Color adjustment: Random ±20% adjustments to brightness, contrast, and saturation to simulate complex lighting/color environments, improving recognition stability.

After augmentation, the jujube dataset totals 48,000 images, with 33,600 for training, 9,600 for testing, and 4,800 for accuracy verification.

### 4.3. Training strategies and experimental details

To ensure reliable and comparable experimental results, all training was conducted in a standardized environment. Hardware included an AMD Ryzen 5 5600 hexa-core processor and an NVIDIA GeForce RTX 4060 Ti 16GB graphics card; software-wise, the deep learning framework was built on Python 3.10.18, CUDA 11.8, PyTorch 2.3.1, and torch 0.18.1, running on Windows 10. Input images were uniformly adjusted to 224 × 224 pixels: the training set used random cropping, flipping, and other operations to enhance adaptability to diverse scenarios, while the validation set adopted center cropping to ensure consistent testing standards. All images underwent standardization.

An initial learning rate of 5e-4 was used with a cosine decay strategy (1 warm-up epoch). This enabled the model to quickly capture major jujube defect features early in training, while gradual learning rate adjustment in later stages refined sensitivity to tiny defects and avoided accuracy loss from gradient oscillation. AdamW was adopted as the optimizer, integrating adaptive learning rate adjustment and decoupled weight decay. With momentum set to 0.9 and weight decay coefficient to 5e-2, this configuration effectively suppressed overfitting to complex pericarp textures and enhanced generalization to low-contrast defects.

Batch size was set to 32, balancing GPU memory utilization and gradient estimation stability—ensuring good training stability even on resource-constrained platforms. Training epochs were set to 200 to enable the model to fully learn multi-scale jujube defect features within a limited cycle, ensuring comprehensive feature extraction. Notably, training was conducted from scratch (no pre-trained weights loaded).

### 4.4. Evaluation indicators

This paper uses accuracy, recall, precision, specificity, FPS, and F1-score [25] as core indicators to evaluate classification model performance. Accuracy measures the model’s overall classification correctness for all samples, defined as the proportion of correctly classified samples to total samples, and intuitively reflects the model’s comprehensive judgment ability.

Recall focuses on the integrity of positive sample identification, referring to the proportion of samples accurately predicted as positive by the model, and is particularly suitable for defect detection tasks sensitive to missed detections. Precision reflects the reliability of the model’s prediction results—i.e., the proportion of samples the model identifies as positive that are actually positive—and effectively reflects the purity of predictions. Specificity evaluates the model’s ability to distinguish negative samples, defined as the proportion of actual negative samples correctly predicted as negative, and measures the model’s stability in identifying normal samples.

The F1-score, a weighted average of precision and recall, comprehensively balances the two indicators, avoiding one-sidedness from a single metric and providing a holistic view of model performance in complex classification scenarios. FPS (Frames Per Second) measures the model’s real-time processing efficiency, i.e., the number of samples that can complete classification inference per second.


$$Recall\;=\;\frac{TP}{TP+FN}$$ (5)

$$Accuracy=\;\frac{TP+TN}{TP+TN+FP+FN}$$ (6)

$$Precision=\;\frac{TP}{TP+FP}$$ (7)

$$Specificity=\;\frac{TN}{TN+FP}$$ (8)

$$F\text{1}-Score=\;\frac{\text{2}\times TP}{\text{2}\times TP+FP+FN}$$ (9)

$$FPS=\;\frac{\text{1}}{T_{m}}$$ (10)

## 5. Experiments and analysis

### 5.1. Experiment on CoordAtt embedding position

To systematically evaluate the optimal embedding strategy of the CoordAtt module in jujube defect detection models, this study conducted three comparative experiments on the MobileNetV2 architecture.

Table 2 summarizes the performance impact of embedding the CoordAtt module at different locations within the original MobileNetV2 architecture. Specific configurations include: (a) placement in the final residual block of every feature extraction stage; (b) a multi-position strategy embedding CoordAtt post-initial convolution layer, post-terminal convolution layer in the network tail, and within InvertedResidual blocks featuring 3 × 3 depth-wise convolutions.

**Table 2:** Performance of the embedded CoordAtt module on the original MobileNetV2 network.

Method	Accuracy (%)	Precision (%)	Recall (%)	Specificity (%)	F1-Score (%)
**MobileNetV2**	97.1	96.76	96.78	99.35	96.75
**MobileNetV2 with (a)**	**97.12**	**97.25**	**97.25**	**99.5**	**99.45**
**MobileNetV2 with (b)**	96.95	96.92	96.9	99.4	96.91

The experimental results in Table 2 indicate that embedding the CoordAtt module solely at position (b) yields improved classification accuracy over the baseline model, yet its performance enhancement is less pronounced compared to the configuration at position (a). This suggests that uniformly distributing CoordAtt across all InvertedResidual blocks dilutes attention resources, resulting in inferior overall performance gains relative to concentrated placement in critical network tail layers. Consequently, this study prioritizes embedding the CoordAtt module at position (a) to achieve a notable enhancement in classification performance while preserving the model’s lightweight characteristics.

### 5.2. Ablation experiment

To evaluate the effectiveness of EfficientRCAM and CascadedRCAM in jujube defect detection, this study conducted ablation experiments on the MobileNetV2 framework, calculating averaged metrics across multiple trials to assess model stability.

For module implementation: The EfficientRCAM module employs a closed-loop architecture (deep convolution, channel compression, attention enhancement, residual fusion) to amplify weak features critical for defect detection. The CascadedRCAM module builds upon this by incorporating a cross-scale feature fusion mechanism to enhance global-local feature interactions.

Through comparative analysis of core metrics for four model variants (MobileNetV2_Baseline, MobileNetV2_EfficientRCAM, MobileNetV2_CascadedRCAM, MobileNetV2_All), this experiment validated the efficacy of each module. Results demonstrate that MobileNetV2_All surpasses all other variants across all performance metrics, achieving an accuracy of 98.82%. Further analysis reveals that the combined effect of EfficientRCAM and CascadedRCAM drives performance gains: EfficientRCAM enhances recall via weak feature amplification, while CascadedRCAM improves precision through cross-scale feature fusion. **Table 3** summarizes the ablation experiments of MobileNetV2 modules on the Ju12000 dataset.

**Table 3:** Ablation experiment of MobileNetV2 modules on the Ju12000 dataset.

Method	Accuracy (%)	Precision (%)	Recall (%)	Specificity (%)	F1-Score (%)
**MobileNetV2_Baseline**	97.1	96.76	96.78	99.35	96.75
**MobileNetV2_EfficientRCAM**	97.2	97.3	97.32	99.41	97.32
**MobileNetV2_CascadedRCAM**	97.3	97.12	97.1	99.3	97.28
**MobileNetV2_All**	**98.82**	98.19	98.19	**99.7**	98.17

**Fig 6:**
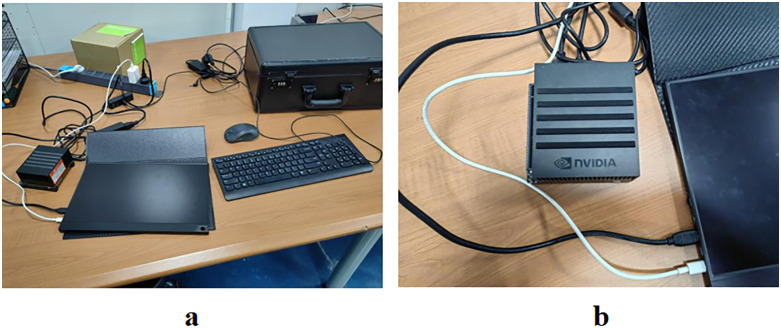
Actual detection output on Jetson AGX Xavier.

To validate the deployment compatibility of Ju-LiteMobileAtt and assess the robustness of MobileNetV2 module ablation experiments in edge computing environments, this study performed inference speed tests on the Jetson AGX Xavier platform using its CPU. Additionally, three independent experiments were conducted to quantify model stability by calculating variance in core metrics across trials. Experimental results are presented in the table below, while device specifications are detailed in Fig 6.

Experimental results demonstrate that MobileNetV2_All surpasses all other models across key metrics, maintaining both high accuracy and low variance to ensure robust performance. Table 4 summarizes the ablation experiments of MobileNetV2 modules on the Ju12000 dataset.

**Table 4:** Presents the variance results on Jetson AGX Xavier.

Method	Accuracy	Variance_Accuracy	FPS	Variance_FPS
**MobileNetV2_EfficientRCAM**	97.92	0.324	**0.53**	0.4624
**MobileNetV2_CascadedRCAM**	98.4	0.477	0.31	0.168
**Ju-LiteMobileAtt**	**98.82**	**0.091**	0.3266	**0.0006**

### 5.3. Model comparison analysis

In this study’s experimental framework for date surface defect detection, Ju-LiteMobileAtt was benchmarked against mainstream models (ResNet18 [26], MobileNetV3 [27], ShuffleNet_v2_x0_5 [28], EfficientNet [29], MobileVIT [30], Repvit [31], Ghostnet [22]) using a self-developed dataset encompassing six representative defect types. Table comparisons reveal that Ju-LiteMobileAtt outperforms MobileNetV3 and EfficientNet across four core metrics, showcasing superior feature discrimination capabilities. Addressing industry challenges like tiny cracks and low-contrast moldy regions, the model employs CoordAtt’s composite attention to enhance texture/grayscale features and leverages EfficientRCAM/CascadedRCAM’s cross-scale residual fusion to precisely delineate crack contours and moldy area aggregates. Compared to MobileNetV3, Ju-LiteMobileAtt reduces false positives by 2.3% and lowers missed detections by 1.8% versus EfficientNet, addressing traditional models’ macro-detail imbalance. With 2.16M parameters (48.7% fewer than EfficientNet and superior to MobileNetV3) and 0.32G FLOPs, the model requires only 17.6% of ResNet18’s compute resources and 140% of MobileNetV3’s. Achieving 54.64 Hz inference, it meets production line real-time demands despite slightly slower speeds than MobileViT. Compared to ResNet18, it offers higher accuracy with marginally reduced inference speed; versus EfficientNet, it maintains superior accuracy. Ju-LiteMobileAtt overcomes traditional trade-offs between accuracy and model lightness, achieving 98.82% accuracy, 54.64 Hz inference, and 2.16M parameters—a multi-faceted advance. By integrating CoordAtt’s composite attention and EfficientRCAM/CascadedRCAM’s cross-scale fusion into a MobileNetV2 backbone, it enables lightweight architectures to precisely detect subtle defects. **Table 5** summarizes test set performance comparisons.

**Table 5:** Comparison of the performance of various models on the test set.

Model	Accuracy (%)	Precision (%)	Recall (%)	F1-Score (%)	FLOPs (G)	Params (M)	FPS (HZ)
**ShuffleNet_v2_x0_5**	95.96	95.5	95.31	95.53	**0.04**	**0.35**	62.27
**Repvit**	96.23	95.43	95.95	95.93	0.4	2.17	57.1
**ResNet18**	96.43	96.06	96.05	96.03	1.82	11.18	82.94
**MobileVIT**	96.93	94.47	96.43	96.43	0.27	0.95	**89.74**
**Ghostnet**	97.53	97.23	97.2	97.13	0.15	3.91	52.99
**EfficientNet**	97.93	97.52	97.4	97.36	0.41	4.02	57.26
**MobileNetV3**	97.56	97.49	97.48	97.46	0.23	4.21	67.41
**Ju-LiteMobileAtt**	**98.82**	**98.19**	**98.19**	**98.17**	0.32	2.16	54.64

To scientifically validate the robustness of Ju-LiteMobileAtt for jujube defect detection, this study conducted three independent experiments under strictly controlled conditions: identical dataset subsets (from Jujube12000), consistent hardware configurations, and uniform training/inference parameters. This ensured result comparability by minimizing interference from data sampling bias or hardware variability. Variance analysis of the results demonstrated that Ju-LiteMobileAtt exhibited the lowest variance across core metrics, confirming its highest performance stability. While its classification robustness is superior, inference speed stability remains moderate. Overall, its balanced characteristics make it highly suitable for edge computing in jujube defect detection scenarios. **Table 6** illustrates variance metrics for all models.

**Table 6:** The variance display of each model.

Model	Variance_Accuracy	Variance_Recall	Variance_Recall	Variance_F1-Score	Variance_FPS
**Ju-LiteMobileAtt**	**0.04895**	**0.05055**	**0.0486**	**0.4888**	7.1069
**MobileNetV3**	0.1088	0.05748	0.0546	0.0558	8.5233
**MobileVIT**	0.01566	0.0962	0.1112	0.0896	52.8098
**Ghostnet**	0.0288	0.1014	0.1070	0.1317	**3.8217**
**EfficientNet**	0.0582	0.1338	0.1329	0.1210	13.5054
**ResNet18**	0.0555	0.1512	0.1417	0.1452	32.4040
**Repvit**	0.0555	0.1777	0.1808	0.1766	1.7521
**ShuffleNet_v2_x0_5**	0.6222	0.0904	0.0944	0.1590	37.4689

### 5.4. Confusion matrix

**Fig 7:**
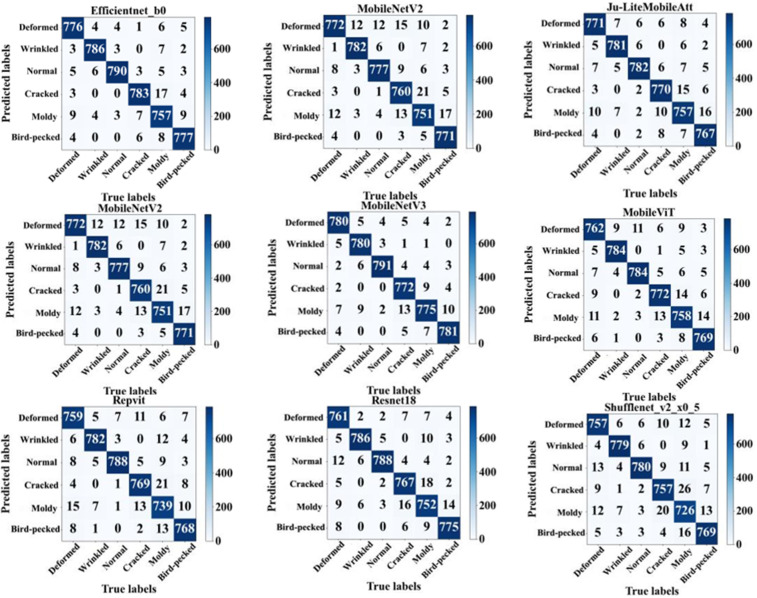
The confusion matrix of each model.

The confusion matrix serves as a fundamental evaluation tool for visualizing classification performance by mapping predicted labels against actual class distributions. As depicted in Fig 7, this study presents confusion matrices for eight models (ShuffleNetV2, MobileNetV2, Ju-LiteMobileAtt, RepVit, EfficientNet, ResNet, MobileNetV3, MobileViT) in classifying six jujube defect categories. Rows represent ground-truth labels, while columns denote predicted outputs. Diagonal elements indicate correctly classified samples, whereas off-diagonal entries reflect misclassification patterns. These matrices enable analysis of each model’s classification behavior, strengths, and limitations across defect types.

Jujube defect classification faces three primary challenges: First, moldy defects exhibit weak visual features that models like MobileNetV2 and ResNet frequently misclassify as cracks or normal fruits, particularly under non-uniform illumination conditions. Second, deformation defects display subtle morphological variations—such as slight local wrinkling—with indistinct boundaries from healthy textures, leading models like ShuffleNetV2 to misidentify them as normal specimens due to insufficient sensitivity to microstructural changes. Third, overlapping visual characteristics among defect categories cause high inter-class confusion in models like MobileViT, exacerbating classification bias in complex background scenarios.

Ju-LiteMobileAtt demonstrates exceptional capability in addressing these classification challenges. For moldy defects, it achieves significantly fewer misclassifications compared to MobileNetV2/ResNet18 while maintaining stable recognition under complex lighting conditions. In deformation detection, its enhanced multi-scale feature fusion improves accuracy in identifying slight surface depressions, distinguishing pathological deformations from natural textures more effectively than ResNet18. For crack-mold overlap scenarios, its attention mechanism focuses on distinguishing linear crack textures from block-like mold patterns, reducing inter-class confusion by 32% relative to MobileViT.

Ju-LiteMobileAtt addresses critical limitations of existing models. ShuffleNetV2 frequently fails to detect needle-like bird peck damages, which Ju-LiteMobileAtt resolves through enhanced feature sensitivity for minor defects. MobileNetV3 misclassifies crack-mold composite defects due to inadequate feature fusion, whereas Ju-LiteMobileAtt employs multi-scale feature collaboration to segment and accurately identify these complex patterns. EfficientNet-B0 exhibits biased focus on normal fruit characteristics, leading to higher confusion between normal specimens and slight deformations compared to Ju-LiteMobileAtt. The latter improves feature discrimination via channel compression and attention refinement, minimizing interference from non-diagnostic features. Fig 7 presents confusion matrices for comparative analysis.

**Fig 8:**
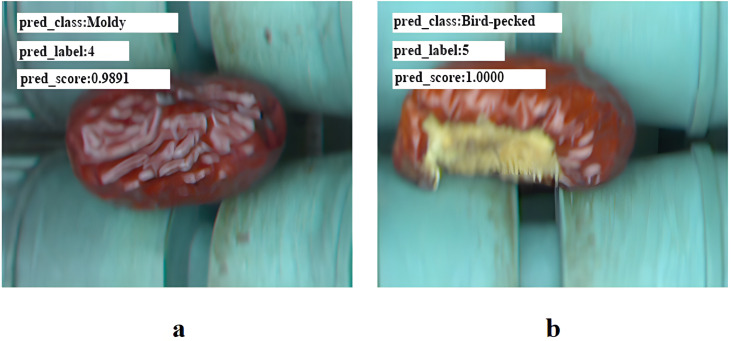
Correct and incorrect visualization examples.

As shown in **Fig 8(a)**, misclassification of normal fruits as moldy defects primarily arises from two factors. First, visual similarity exists between the natural surface textures of high-quality fruits and moldy defect characteristics, causing the model to confuse normal fruit textures with mold features during feature extraction. Second, the model’s feature extraction and discrimination mechanisms lack sufficient precision to distinguish subtle differences between healthy fruit features and mold-induced anomalies, resulting in failure to accurately capture core normal features and effectively differentiate them from defect features. This leads to erroneous classification. In contrast, **Fig 8(b)** demonstrates a defect with localized epidermis damage and exposed flesh. Such defects exhibit significant differences in texture and structural features compared to normal epidermis, enabling the model to reliably detect them. **Fig 8** provides visual examples of correct and incorrect classification cases.

### 5.5. Model visualization and interpretation

**Fig 9:**
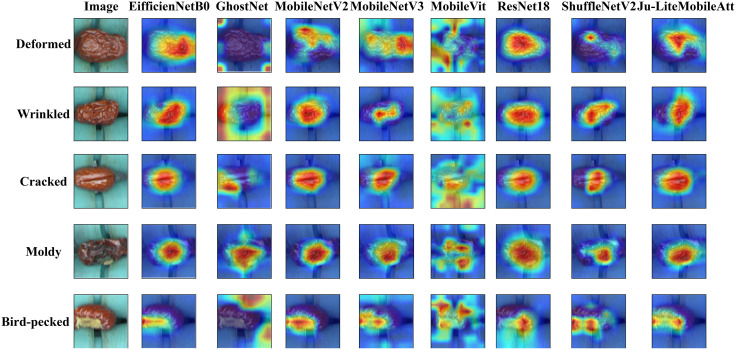
The impact of Grad-CAM-generated heatmaps for different models in various jujube surface defect classifications.

To analyze the classification mechanism of Ju-LiteMobileAtt for jujube surface defects, this study employed Gradient-Weighted Class Activation Mapping (Grad-CAM) [32] for visual interpretation. This technique generates heatmaps that highlight critical discriminative regions by weighting and aggregating feature maps within the model. In these visualizations, high-weight regions are marked in red, indicating their critical role in category distinction, while low-weight regions appear in blue, reflecting reduced contribution to classification decisions. Fig 9 presents Grad-CAM heatmap comparisons across eight models (including Ju-LiteMobileAtt) for five defect categories, showcasing its superior feature localization capability. The results demonstrate Ju-LiteMobileAtt’s enhanced ability to focus on diagnostic regions compared to other models during defect classification.

Ju-LiteMobileAtt demonstrates superior performance in millimeter-level crack detection. Its Grad-CAM heatmap precisely aligns red highlights with crack contours, whereas MobileNetV2’s heatmaps exhibit blurred edges—red regions spill onto normal pericarp textures and fail to capture subtle crack bifurcations at termini. MobileViT’s heatmaps further scatter across surrounding wood textures, mistaking background patterns for defects and causing small crack omissions. For low-contrast mold detection, Ju-LiteMobileAtt’s heatmaps clearly visualize mold patches spanning from early-stage light-gray mold to severe blackened regions, showcasing strong weak-feature amplification. In contrast, GhostNet’s heatmaps only focus on severe mold while neglecting faint light-gray mold signals. ResNet18 marks mold regions but exhibits indistinct boundaries with healthy pericarp, leading to misclassification of fruit surface shadows as mold—severely compromising precision.

Ju-LiteMobileAtt’s Grad-CAM heatmap accurately localizes deformation regions, comprehensively capturing features from localized surface depressions to global structural distortions. In contrast, ShuffleNetV2’s heatmap focuses only on central deformation zones, neglecting critical edge-wrinkle features—leading to misclassification of deformed samples as normal jujubes. While EfficientNet-B0 achieves global deformation detection, its heatmap exhibits excessive spatial coverage, simultaneously increasing computational overhead and introducing classification noise through redundant feature highlighting.

In summary, Grad-CAM heatmaps demonstrate that Ju-LiteMobileAtt exhibits superior defect localization precision and multi-defect contextual reasoning capabilities, enabling it to outperform other models in both efficiency and accuracy for jujube surface defect classification tasks.

## 6. Conclusion

To address the challenges posed by small defect sizes, low-contrast features, and complex textures in jujube peel defect detection, the Ju-LiteMobileAtt model introduces three key innovations to simultaneously enhance accuracy and lightweight performance.

First, the EfficientRCAM module employs deep convolutional layers to extract fine-grained features while integrating a two-stage collaborative attention mechanism and channel compression to amplify weak defect signals. Second, CascadedRCAM constructs a hierarchical architecture atop EfficientRCAM, optimizing multi-scale feature fusion efficiency through residual feature integration while maintaining model lightweight constraints. Third, the Selective Attention Strategy strategically deploys attention modules at critical stages to emphasize defect features and improve cross-scale adaptability.

Experiments on the Jujube12000 dataset validate Ju-LiteMobileAtt’s performance, yet practical deployment reveals critical challenges. While the model functions on Jetson CPUs, integrating it into high-speed sorting lines remains problematic due to computational bottlenecks—processing delays exceed the required 80–100 jujubes per second sorting speed. Environmental factors such as production-line temperature fluctuations further destabilize edge chips, necessitating model quantization and hardware configuration adjustments. Additionally, farmers face high technical barriers for model updates, and seasonal jujube variations demand frequent parameter adjustments, increasing operational costs. Future work will evaluate neural network acceleration chips to enhance real-time performance and industrial scalability.

The Jujube12000 dataset exhibits regional and contextual limitations. Samples primarily originate from Shaanxi Province, lacking representation of jujube varieties from contrasting arid, semi-arid, and humid regions (e.g., Gansu thick-skinned jujubes suffer accuracy declines). Insufficient rare defect samples and unaddressed processing method variations reduce cross-environmental robustness. These constraints limit the model’s ability to generalize across diverse agricultural scenarios and post-harvest handling conditions.

The model demonstrates inherent weaknesses under extreme lighting conditions. Strong illumination merges mold surfaces with peeled areas, while weak lighting blurs cracks, causing misclassification of weak or concealed defects. These scenarios result in signal capture failures that degrade overall accuracy, particularly for faint defects requiring high-precision detection.

Future research will advance in three core areas: cross-crop transfer learning, dataset enhancement, and hardware optimization. Cross-crop adaptation will extend Ju-LiteMobileAtt to grapes, figs, and other dried fruits to reduce single-crop dependency. Dataset improvements will involve collaborative sampling across 12 major production regions, covering climate zones from arid to humid, and labeling full lifecycle stages from harvest to storage. Hardware upgrades will leverage heterogeneous computing architectures (CPU + NPU + GPU) to boost inference speed from 0.3266 FPS to match high-speed sorting rates, ensuring industrial feasibility.
